# Baseline cross-sectional imaging of locally advanced high-risk breast cancer facilitates highly customized radiation therapy in surgically inaccessible anatomical areas

**DOI:** 10.3389/fonc.2025.1556122

**Published:** 2025-02-27

**Authors:** Tomasz Borowiec, Rafał Matkowski, Bożena Cybulska-Stopa, Tomasz Kuniej, Andrzej Kołodziejczyk, Dorota Dupla, Adam Maciejczyk

**Affiliations:** ^1^ Lower Silesian Oncology, Pulmonology and Hematology Center, Wroclaw, Poland; ^2^ Department of Oncology, Wroclaw Medical University, Wroclaw, Poland; ^3^ Department of Hematology and Oncology, Faculty of Medicine, Wroclaw University of Science and Technology, Wrocław, Poland

**Keywords:** breast cancer, high-risk patients, primary systemic therapy, pre-treatment imaging, treatment individualization

## Abstract

**Background:**

Routine medical imaging used for preliminary breast cancer workup, such as mammography (MMG) and ultrasound (US), has limited utility for radiation oncologists. We hypothesized that the inclusion of cross-sectional imaging (CT scan or PET-CT) prior to primary systemic therapy (PST) would improve clinical staging accuracy and facilitate customized postoperative radiation therapy planning. Therefore, this study aimed to compare the standard baseline imaging with extended radiological staging.

**Methods:**

To assess our hypothesis, we performed a prospective, single-center study that included 132 participants who were recruited from October 2015 to March 2020. We quantified the value of cross-sectional imaging compared to those of MMG and US. Descriptive statistics, the Friedman and chi-square tests were performed, and p < 0.05 was considered significant.

**Results:**

Patients were grouped into two cohorts: the CT scan cohort (n = 87) and the PET-CT cohort (n = 43). A comparison of the value of cross-sectional imaging with those of MMG and US revealed that staging and radiation planning were altered by this additional procedure. The originally determined disease stage changed in 36.8% and 51.2% of cases in the first and second groups, respectively. The consistency between the assessment of involved axillary lymph nodes using imaging (cN) and the postoperative pathology report (pN) were evaluated. In most cases, clinical and pathological evaluation were consistent, with χ2(1) = 18.98; p < 0.001 for CT scan, and χ2(1) = 6.41; p = 0.03 for PET-CT.

**Conclusions:**

Cross-sectional imaging is recommended for patients with locally advanced high-risk breast cancer. A highly customized radiation therapy, including a dose boost, was administered in nine patients with affected lymph nodes that were surgically inaccessible. This procedure was facilitated by extended radiological staging.

## 1 Introduction

Breast cancer is the most common malignancy among women worldwide. In the United States, over 290,000 new cases are diagnosed annually (1). The treatment of patients with breast cancer has evolved to a multidisciplinary approach. In the surgical treatment of breast cancer, breast-conserving therapy (BCT) is increasingly used. Extensive axillary node dissections are becoming less common. Two anatomical nodal areas seem to be particularly interesting. These are the supraclavicular region and the internal mammary (IM) nodes. Both of these are not routinely operated on. In case of involvement of IM nodes minimally invasive surgical techniques such as video-assisted thoracoscopic surgery (VATS) are safe, but there is a lack of data on whether patients actually benefit clinically from such a procedure (2). In patients with supraclavicular node involvement, radiotherapy alone seems to give better results than surgery combined with radiotherapy (3). Metastases in the IM nodes are usually not an isolated phenomenon but coexist with axillary nodes involvement. Based on the available knowledge, it is difficult to clearly recommend which imaging tests should be performed in in such cases (4).

**Figure 1:**
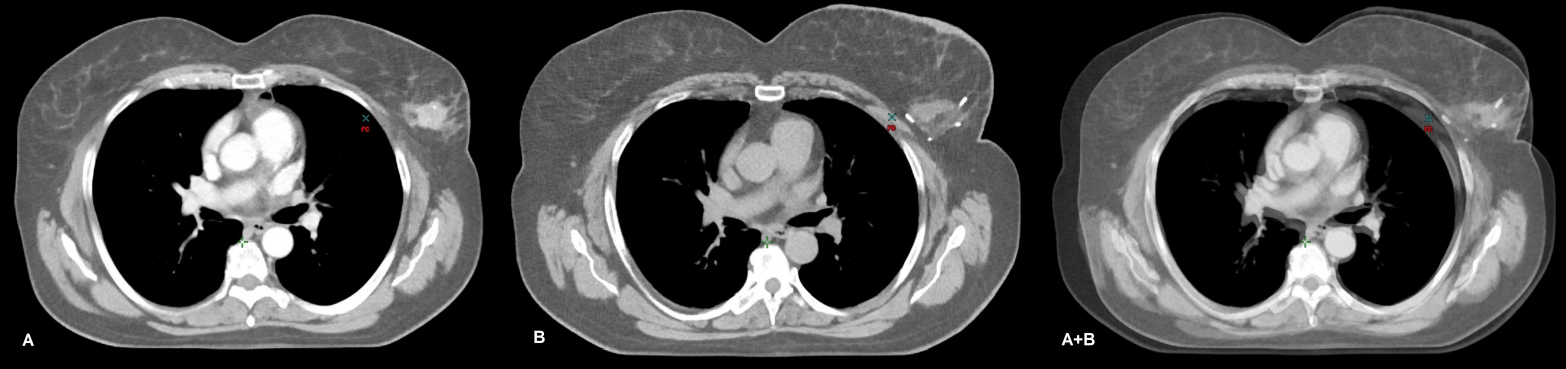
CT images of a participant in the CT scan cohort with cT2N0M0, G3 triple-negative breast cancer (TNBC) of the left breast. A contrast-enhanced focus is observed on the CT scan performed before systemic therapy **(A)**. The planning CT of the same patient demonstrates the breast tumor bed and tracers inserted by the surgeon **(B)**. Both images were taken in the same position. Although slight variations were observed in the respiratory phase, it does not affect the generation of fairly reliable fusion image by overlapping the two CT images (A+B). This approach may be facilitated while contouring the boost volume (increased irradiation dose).

**Figure 2:**
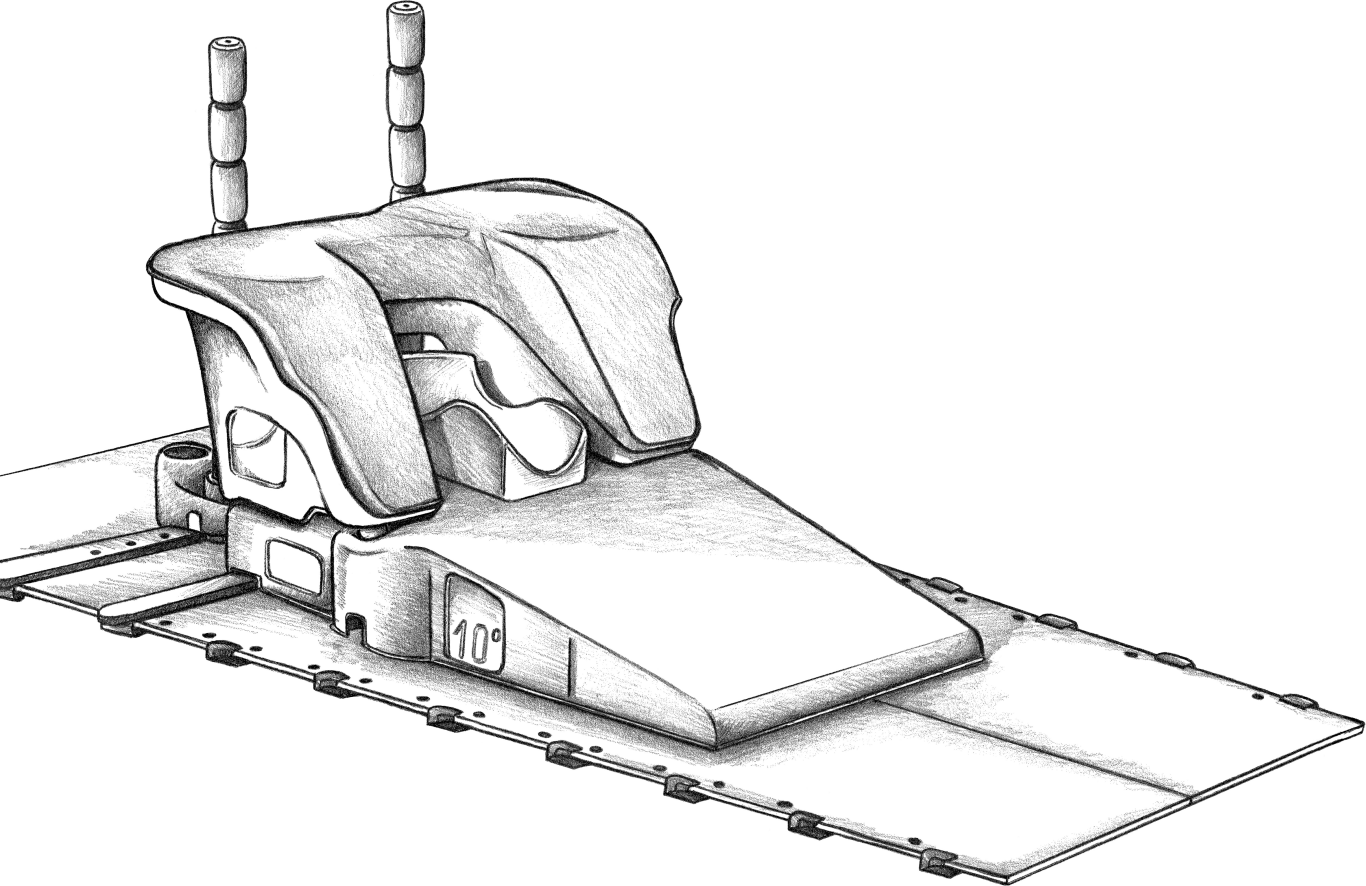
Orfit-AIO system was used for positioning. The layout can be customized based on various factors, such as anatomical conditions, breast size, and the patient’s fitness. Most of the sets enable the selection of appropriate back support and adjustment of the location of the shoulder immobilization. The positional information can be easily stored for future reference, allowing for the recreation of the same body posture during subsequent CT scans or PET-CT.

Currently, initial systemic therapy is administered in most triple-negative breast cancer (TNBC) and HER2-positive cases to achieve pathological complete response (pCR). Irradiation is performed after surgical treatment to improve local control and overall survival in early and advanced breast cancer (5). When significant clinical response to systemic therapy is achieved, areas at risk of persistent disease (such as enlarged supraclavicular nodes) will not be evident on post-operative computed tomography (CT) scans that are used for treatment planning. Standard imaging techniques for diagnosis and staging of breast cancer, including mammography (MMG), ultrasound (US), and magnetic resonance imaging (MRI), have limited utility in personalizing radiation therapy (RT); therefore, planning systems are mainly based on CT images. MMG does not adequately visualize lymph nodes; moreover, it is more suitable for the evaluation of the primary foci in the low mammographic density breasts of postmenopausal women than in the glandular breasts of younger patients (6, 7), who are relatively frequently qualified for primary systemic therapy. Advanced RT techniques, such as intensity modulated radiation therapy (IMRT) require defining the desired dose to all target and non-target tissues on each slice of the planning CT. High-quality cross-sectional imaging that allows 3D visualizations, such as CT scans or positron emission tomography (PET-CT), are a necessary part of the procedure. Despite available data suggesting the utility of CT scan and PET-CT in breast cancer, they are not routinely performed (8–10). Cross-sectional imaging obtained before systemic therapy and surgery, followed by planning CT images, offers numerous possibilities for RT customization, such as increased dose in the non-operated anatomical area, especially where pathological lymph nodes were observed (e.g. supraclavicular region or internal mammary lymph nodes). Additionally, it can aid in diagnosing oligometastatic disease and can facilitate the application of stereotactic body radiation therapy (SBRT). Scans performed before cancer treatment and planning CT done postoperatively could be compared or superimposed on each other in a process referred to as image fusion, providing a tool for RT individualization (**Figure 1**). In our study, a radiation immobilization device was used to position patients during imaging (**Figure 2**). The aim of this single-center study was to compare standard baseline imaging with extended radiological staging in patients who are qualified for primary systemic therapy. Furthermore, we aimed to investigate whether CT scans and PET-CT can reliably visualize the primary focus in the breast as well as pathological lymph nodes in the axilla, and to determine whether the nodes that were recognized as being affected actually contained metastases by assessing the correlation between postoperative pathological reports and imaging results. Additionally, we analyzed whether extended radiological staging had an additional diagnostic value. The secondary objective of the study was to investigate how often the multidisciplinary team (MDT) modified the originally planned treatment strategy after receiving an additional examination result.

## 2 Materials and methods

This was a prospective, cross-sectional, observational study. Data was collected from patients’ medical records. Eligible patients comprised those aged ≥18 years with an established diagnosis of invasive breast adenocarcinoma after core needle biopsy. Assessment of histological grade and immunohistochemistry (IHC) evaluation of estrogen receptor (ER), progesterone receptor (PgR), Ki67, and human epidermal growth factor receptor 2 (HER2) status was required in the histopathology report. If the HER2 score was borderline, fluorescence *in situ* hybridization (FISH) was performed. Patients with an Eastern Co-operative Oncology Group Performance Status of 0–2, no distant metastases, and no clinically significant renal failure were recruited. All participants were selected for systemic preoperative therapy by a MDT at the Breast Unit of the Lower Silesian Oncology, Pulmonology, and Hematology Center in Wroclaw, Poland. After performing preliminary radiological staging according to the recommendations of the Polish Society of Clinical Oncology (bilateral MMG, breast US, and chest X-ray (CXR) regardless of the stage, abdominal cavity imaging using US and/or CT scan, and bone scintigraphy in CS III) (11), all eligible patients were offered an extended workup. The current Polish national recommendations indicate that additional imaging studies are optional; moreover, routine inclusion of chest cross-sectional imaging (CT scan or PET-CT) prior to primary systemic therapy (PST) is not commonly practiced. Considering the estimated risk of invasion and metastasis, the decision to perform one of the imaging modalities was made by the MDT. High-risk patients underwent PET-CT without strict criteria for selecting additional imaging modality. Two cohorts of patients were analyzed separately: a CT scan was performed in the first cohort, while a PET-CT was performed in the second. All imaging and treatment results used in the study were considered a part of the patients’ diagnostic and therapeutic scheme. Overall, 132 participants were recruited between October 2015 and March 2020; however, after detailed verification, two patients were excluded. In one case, a surgical biopsy of the breast tumor eventually led to a tumorectomy, and the breast lesion could not be measured. In the other case, the assumed period of 2 weeks from the beginning of systemic therapy to the CT scan was exceeded. Further analyses included 130 participants (128 female and two male patients). All PET-CT examinations were performed before treatment, and a CT scan was allowed up to 2 weeks after starting systemic therapy, assuming that during this time there would be no significant tumor shrinkage in most patients. The recruitment process was interrupted during the COVID-19 pandemic, as participation required an additional clinic visit, which could increase the risk of coronavirus transmission. The last CT scan was performed on March 12, 2020, while the collection of COVID-19 statistics in Poland began on March 14, 2020 (according to the COVID-19 Data Repository of the Center for Systems Science and Engineering (CSSE) at Johns Hopkins University, there were 35 COVID-19 cases in Poland at that time). Thus, it can be concluded that CT scan and PET-CT results were unaffected by COVID-19 related bias, regarding both the lung parenchyma and potential vaccination-related lymphadenopathy (12). All scans were performed with the patient in the supine treatment position, were supervised by a radiation oncologist and experienced technicians; this position was also suited to the patient’s anatomical structure. The prone position is not optimal if lymph node radiation is planned, and current guidelines and contouring atlases, which are helpful in daily practice, recommend the supine position (13–15). All CT scans were assessed by an experienced radiologist, and PET-CT were assessed by a nuclear medicine specialist. Measurements of tumor sizes were also made by the above-mentioned professionals. CT was performed using an intravenous contrast agent and PET-CT using fluorodeoxyglucose (2-[18F] FDG) as the radio-active tracer. The cross-sectional imaging result was attached to the patient’s medical history and analyzed by the MDT.

Statistical analysis was performed using IBM SPSS Statistics 25.0 software (IBM Corp., Armonk, N.Y., USA). The Friedman test was used to evaluate the difference between the largest dimensions of the breast tumors in alternative medical imaging methods. Pairwise comparisons were performed using the *post-hoc* Dunn’s test. The following descriptive statistics were considered: mean, standard deviation, median, minimum, maximum, and the first and third quartiles. The distribution of the analyzed variables was presented in detail. Pair collation was made based on the median. We compared the largest dimension of the largest breast tumor focus (T/mm), between the standard MMG, US, and CT scans, as well as between MMG, US, and PET-CT. Chi-square test was applied to estimate the distribution of the variables pN+ (involved axillary nodes based on the histopathology report) and cN+ (involved axillary nodes based on imaging). Consistency between the clinical assessment using imaging and the pathology report was estimated separately in the CT scan cohort and the PET-CT cohort. The rate of patients was calculated in terms of an additional diagnostic value of extended radiological staging, which resulted in a modification of the clinical stage or change in the management strategy. The level of statistical significance was set at p < 0.05.

## 3 Results

### 3.1 Patient demographics and tumor characteristics

The age of the participants was similar in CT scan and PET-CT cohorts, with a median of 51 and 50 years, respectively. High-grade G3 histologic tumors were the most common and have been confirmed in 55.1% of cases in the CT scan cohort and in 62.8% in the PET-CT cohort; the G1 grade was observed in only one participant. High Ki67 expression was predominant, with a median of 43.5 (7–90) in the CT scan cohort and 52.5 (2–90) in the PET-CT cohort (**Table 1**). Participants who qualified for PET-CT, according to the assessment of the MDT, had a higher risk of cancer spread and were, in several cases, originally non-operable. A cT4 clinical tumor stage was established in 39.5% of patients in this group. In the CT scan cohort, several patients (58.6%) had cT2 features. The most common subtype, based on the receptor profile and Ki67 rate, according to the St. Gallen surrogate classification for breast cancer (16), was luminal-B-like HER2 negative. Endocrine therapy was rarely used as a form of preoperative treatment; however, it was used in three patients in the PET-CT cohort and in eight patients in the CT scan cohort. In 91.5% of the participants, PST was based on multi-drug chemotherapy. Anti-Her2 targeted therapy was administered when indicated. Optimal systemic therapy was selected by the MDT. The rate of pathologic complete response was rather low, slightly exceeding 22% in both cohorts, due to a high representation of patients with T4 and N3 features and the dominant subtype being Luminal B (HER2-negative) (17).

**Table 1:** Patients’ baseline characteristics.

		CT scan cohort (n=87)	PET-CT cohort (n=43)
Median age (years)		51 (25–80)	50 (24–74)
Sex	Women	85 (97.7%)	43 (100%)
	Men	2 (2.3%)	0
Clinical tumour stage (cT)	cT1	3 (3.4%)	0
	cT2	51 (58.6%)	16 (37.2%)
	cT3	25 (28.7%)	10 (23.3%)
	cT4	8 (9.2%)	17 (39.5%)
Clinical nodal stage (cN)	cN0	24 (27.6%)	7 (16.3%)
	cN1	26 (29.9%)	8 (18.6%)
	cN2	26 (29.9%)	14 (32.6%)
	cN3	11 (12.6%)	14 (32.6%)
Histologic grade	G1	0	1 (2.3%)
	G2	37 (42.5%)	14 (32.6%)
	G3	48 (55.1%)	27 (62.8%)
	not established	2 (2.3%)	1 (2.3%)
Median Ki67		43,5 (7–90)	52,5 (2–90)
St. Gallen surrogate classification for breast cancer	Luminal A-like	5 (5.7%)	4 (9.3%)
	Luminal B-like (HER2-negative)	34 (39.1%)	18 (41.9%)
	Luminal B-like (HER2-positive)	11 (12.6%)	9 (20.9%)
	HER2-positive (non-luminal)	8 (9.2%)	1 (2.3%)
	Triple-negative	29 (33.3%)	11 (25.6%)
Definitive surgery		82 (94.3%)	35 (81.4%)
No surgery		5 (5.7%)	8 (18.6%)
Pathologic complete response (pCR)		18 (22%)	8 (22.9%)
No pathologic complete response		64 (78%)	27 (77.1%)

Histologic grade rated on Scarf-Bloom-Richardson Grading System, Nottingham Modification. Abbreviations: CT, computed tomography; PET-CT, positron emission tomography.

### 3.2 Tumor size measurements

The largest dimensions in millimeters (T/mm) of breast tumors measured using alternative medical imaging modalities were compared (**Table 2**). In cases of multifocal tumors, the largest lesion was assessed; the Friedman test was used to evaluate the differences. Only the dimensions evaluated using US were statistically significantly smaller (p = 0.02) than those evaluated using CT scan. However, in both cohorts, US and MMG were performed before the diagnosis was confirmed using biopsy, while CT scan was performed after the initial workup and evaluation by the MDT. Furthermore, disease progression over a period of several weeks may have occurred in some patients. In PET-CT cohort, the measurement of the focus in the breast was made in low-dose CT (LDCT), which is a component of the PET-CT scan. When the dimensions obtained from PET-CT were compared with those obtained from US and MMG, there was no statistically significant difference in the measurements (p = 0.9).

**Table 2:** Largest contiguous dimension of the tumour focus in alternative imaging methods.

CT scan cohort	M	Me	SD	Min	Max	Q1	Q3
T/mm MMG	40.79	37.5	21.88	10	130	25	51.25
T/mm US	38.51	38	13.95	11	75	29	46
T/mm CT scan	43.48	40	19.2	13	115	30	54
PET-CT cohort							
T/mm MMG	44.78	35	26.93	12	100	27	55
T/mm US	44.72	40	21.93	14	100	27	56
T/mm PET-CT	46.32	37.5	26.43	12	120	29.75	56

In case of multifocal tumours, the largest lesion (T) was taken into account. According to the criteria for determining T category in TNM Staging System For Breast Cancer, by American Joint Committee on Cancer (AJCC).

MMG, mammography; US, ultrasound; CT scan, computed tomography; PET-CT, positron emission tomography; M, mean; Me, median; SD, standard deviation; Min, minimum; Max, maximum; Q1, first quartile; Q3, third quartile.

### 3.3 Baseline lymph node evaluation and consistency between the clinical assessment of lymph nodes with the postoperative pathology report

Unambiguous lymph node assessments were obtained using all imaging techniques, with clear differentiation between pathological (cN+) and non-involved (cN-) nodes as shown in **Table 3**. Following the exclusion of unoperated patients and those who reached pCR (both in the primary focus and in axillary nodes), the consistency between the clinical assessment of axillary lymph nodes (cN) using CT scan and US with the postoperative pathology report (pN) was evaluated (**Table 4**). This analysis is reliable because of the large cohort of participants with only a partial response, including 64 in the CT scan cohort and 27 in the PET-CT cohort. In 95.3% of node-positive cases, there were consistencies between microscopic and CT scan evaluations. In two cases (4.7%), lymph nodes were assessed on CT scan as not involved; however, histological examination revealed that they contained metastases. Similar results were obtained after comparing the clinical evaluation of lymph nodes using US and the postoperative pathology report. In 92.5% of node-positive cases, pathology reports and lymph node evaluations in US were consistent; however, in three cases (7.5%), lymph nodes were assessed as not involved using US but were found to contain metastases (**Table 4** **).** This suggests that the diagnostic values of CT scan and US are similar in terms of lymph node assessment. Chi-square test confirmed consistency between the clinical evaluation of involved lymph nodes using imaging (cN+) and the pathology report (pN+); for the CT scan cohort the result was χ2(1) = 18.98; p < 0.001. All histopathological reports were analyzed postoperatively after the patients had received systemic treatment. It can be assumed that in some cases nodal pCR with a simultaneous lack of pCR in the primary focus had occurred. Moreover, node-only pCR occurs approximately twice as often as breast-only pCR (18). Migration of patients from the group cN+ to the pN- group is the expected effect of PST. Biopsy confirmation of lymph node metastases in all patients before systemic therapy is not routinely practiced. In cases of massive lymph node involvements that are visible on US and clinical examination (cN2 and cN3 patients), a biopsy is usually not justified. A small percentage of patients may also experience disease progression during systemic therapy. Therefore, assessments of false negative and false positive rates of axillary status were abandoned. Mirror analysis was also performed in the PET-CT cohort (**Table 5**). The clinical stage of patients who qualified for PET-CT was more advanced. Notably, there was no discordance between the nodes assessed as uninvolved by PET-CT and the postoperative pathology report. Although this cohort was smaller, the result of the Chi-square test confirmed the consistency between the clinical assessment of involved lymph nodes using imaging (cN+) and the pathology report (pN+), with χ2(1) = 6.41; p = 0.03 for PET-CT.

**Table 3:** Accuracy of imaging modalities in lymph node evaluation among patients without pathologic complete response.

No pathologic complete response	CT scan cohort (n=64)
Unequivocal lymph node evaluation by CT scan	60 (94%)
Dubious lymph node evaluation by CT scan	4 (6%)
Unequivocal lymph node evaluation by US	59 (92%)
Dubious lymph node evaluation by US	5 (8%)
No pathologic complete response	PET-CT cohort (n=27)
Unequivocal lymph node evaluation by PET-CT	26 (96%)
Dubious lymph node evaluation by PET-CT	1 (4%)
Unequivocal lymph node evaluation by US	26 (96%)
Dubious lymph node evaluation by US	1 (4%)

An unequivocal result was the one in which the nodes were recognized as pathological (cN+) or not involved (cN-). Results that reported suspicious or enlarged nodes were reckoned as dubious.

CT, computed tomography; PET-CT, positron emission tomography; US, ultrasound.

**Table 4:** Consistency between clinical assessment of axillary lymph nodes (cN) by baseline CT scan, US, and the postoperative pathology report (pN).

Imaging vs pathology report	cN evaluated by CT scan			
	cN-(n = 11)		cN+ (n = 49)	
	n	%	n	%
pN- (n = 17)	9	52.9	8	47.1
pN+ (n = 43)	2	4.7	41	95.3
Imaging vs pathology report	cN evaluated by US			
	cN- (n = 16)		cN+ (n = 43)	
pN- (n = 19)	13	68.4	6	31.6
pN+ (n = 40)	3	7.5	37	92.5

Only patients with no pathologic complete response and unequivocal lymph node evaluation by imaging were included in this analysis (refer to **Table 3**).

CT, computed tomography; PET-CT, positron emission tomography; US, ultrasound.

**Table 5:** Consistency between clinical assessment of axillary lymph nodes (cN) by baseline PET-CT, US, and the postoperative pathology report (pN).

Imaging vs pathology report	cN evaluated by PET-CT			
	cN- (n = 3)		cN+ (n = 23)	
	n	%	n	%
pN- (n = 9)	3	33.3	6	66.7
pN+ (n = 17)	0	0	17	100
Imaging vs pathology report	cN evaluated by US			
	cN- (n = 5)		cN+ (n = 21)	
pN- (n = 8)	5	62.5	3	37.5
pN+ (n = 18)	0	0	18	100

Only patients with no pathologic complete response and unequivocal lymph node evaluation by imaging were included in this analysis (refer to the **Table 3**).

CT, computed tomography; PET-CT, positron emission tomography; US, ultrasound.

### 3.4 Added value of extended radiological staging

We also analyzed whether a CT scan or PET-CT had any additional diagnostic value compared to the standard initial workup. The results confirmed that in 49.4% of the participants, clinically significant information was obtained from CT scans. Internal mammary lymph node involvement was also detected in six patients. In two cases, the supraclavicular lymph nodes were found outside the anatomical boundaries suggested by the ESTRO guidelines for the delineation of lymph nodal areas. In one participant, CT scan findings aided in identifying a previously undiagnosed heart pathology (an interatrial thrombus). Involved lymph nodes in the mediastinum were found in one participant (M1 feature). In ten cases, CT scans revealed satellite foci within the involved breast, and in five cases, pectoral muscle infiltrations were identified. In the PET-CT cohort, one-fourth of the cases ended with modifications of the originally planned treatment strategy, and in 51.2% of cases, the originally determined disease stage changed. PET-CT confirmed the multifocal metastasis in seven patients and was helpful in diagnosing oligometastatic disease in two participants (**Table 6**).

**Table 6:** Impact of the CT scan and PET-CT on the multidisciplinary team’s decision.

CT scan cohort (n=87)	n	%
Management strategy modification	9	10.3
Clinical stage shift	32	36.8
Other clinically significant findings	2	2.3
No added value of extended radiological staging	44	50.6
PET-CT cohort (n=43)	n	%
Management strategy modification	11	25.6
Clinical stage shift	22	51.2
No added value of extended radiological staging	10	23.3

Modification of the management strategy was understood as withdrawal of surgery or personalized radiation therapy (e.g., boost within the internal mammary lymph nodes or stereotactic body radiation therapy [SBRT] in oligometastatic disease).

CT, computed tomography; PET-CT, positron emission tomography.

## 4 Discussion

**Figure 3:**
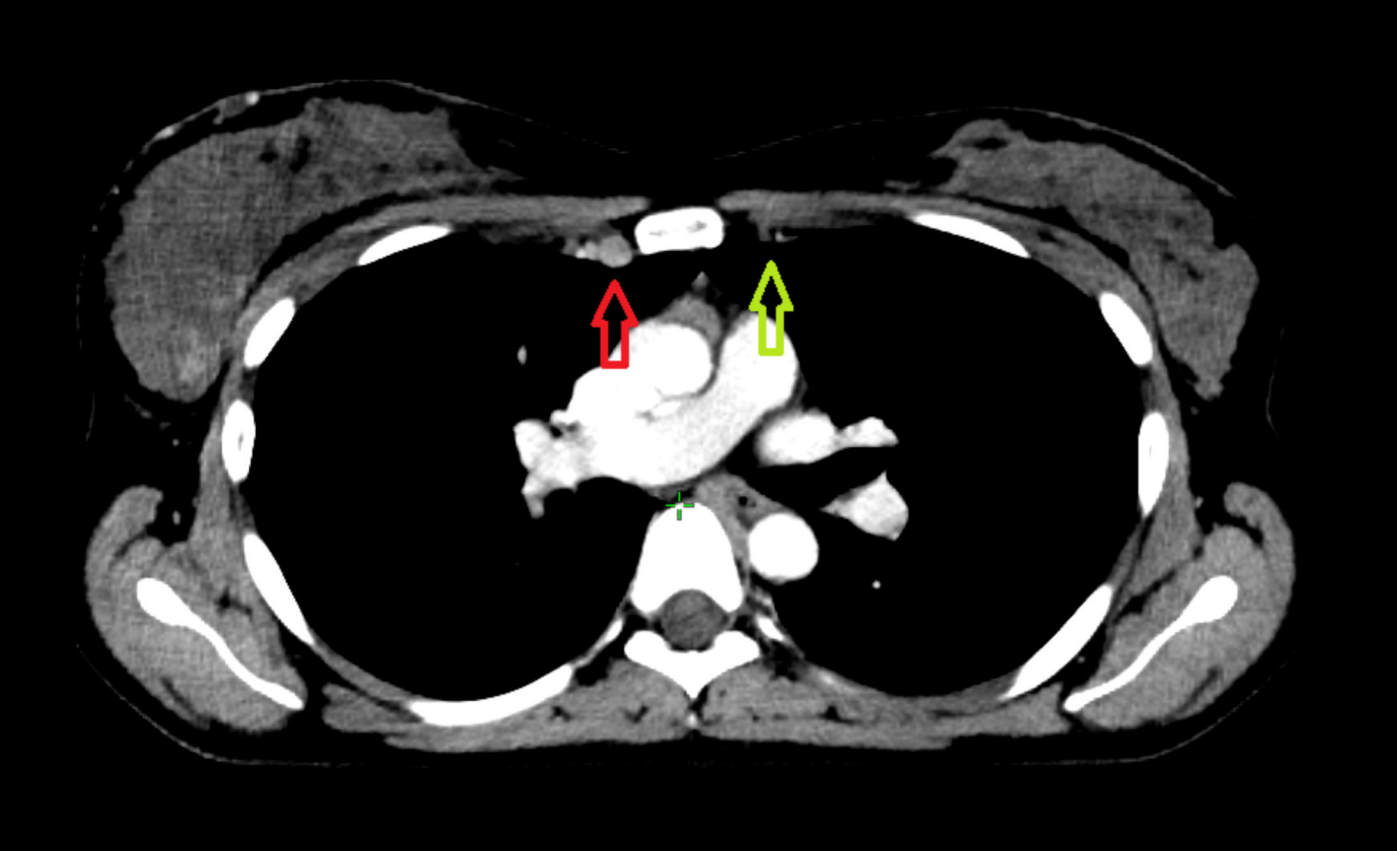
Involved internal mammary nodes, indicated by the red arrow, are difficult to visualize. For comparison, the internal mammary vessels on the contralateral uninvolved side are indicated by the green arrow.

**Figure 4:**
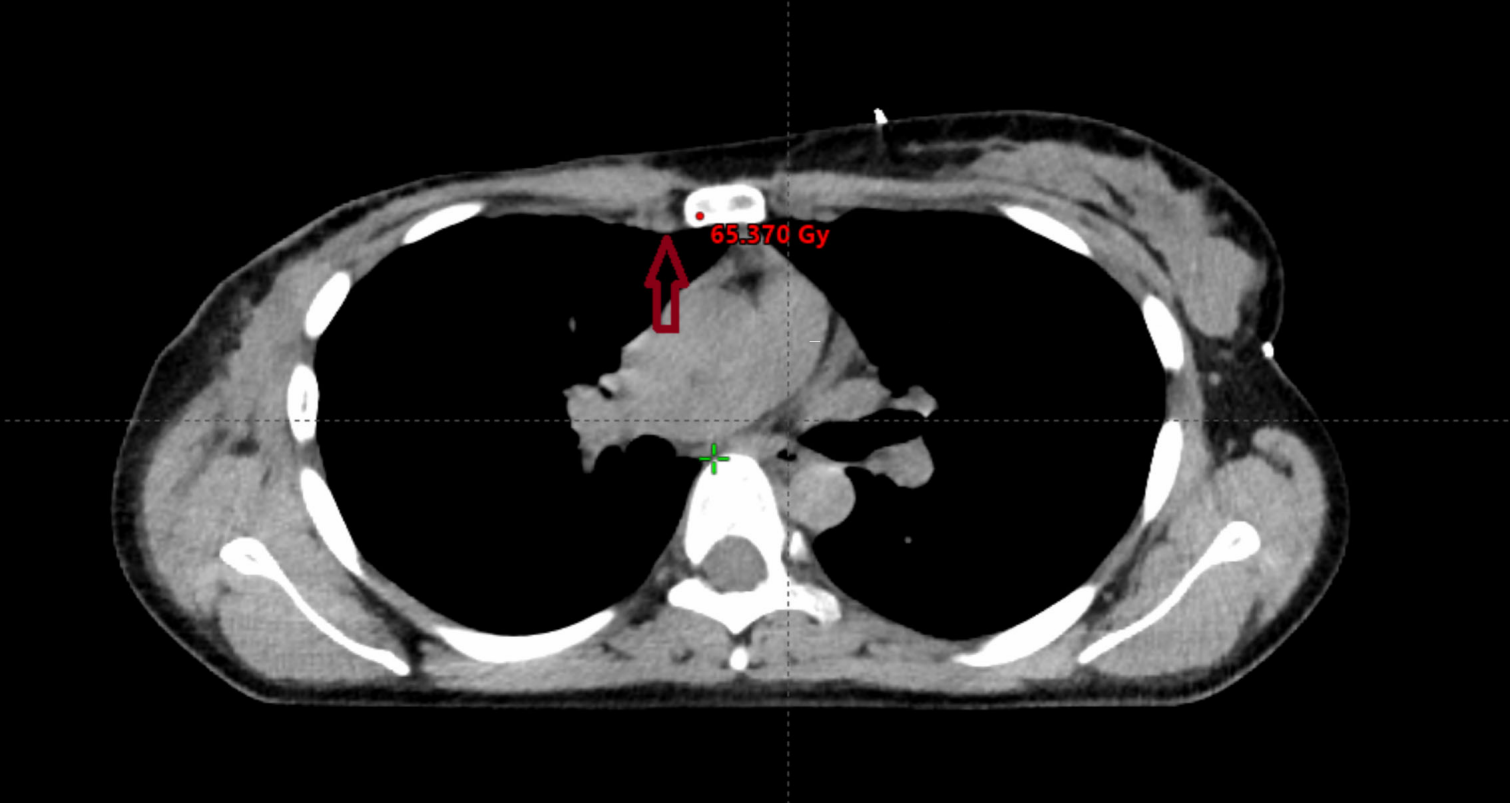
If primary systemic treatment (PST) is effective, remission will occur. On standard planning CT performed without intravenous contrast agent, the involved nodes may temporarily become almost invisible (dark red arrow). This image is from the same patient whose images are in the **Figure 3** after systemic treatment and mastectomy.

**Figure 5:**
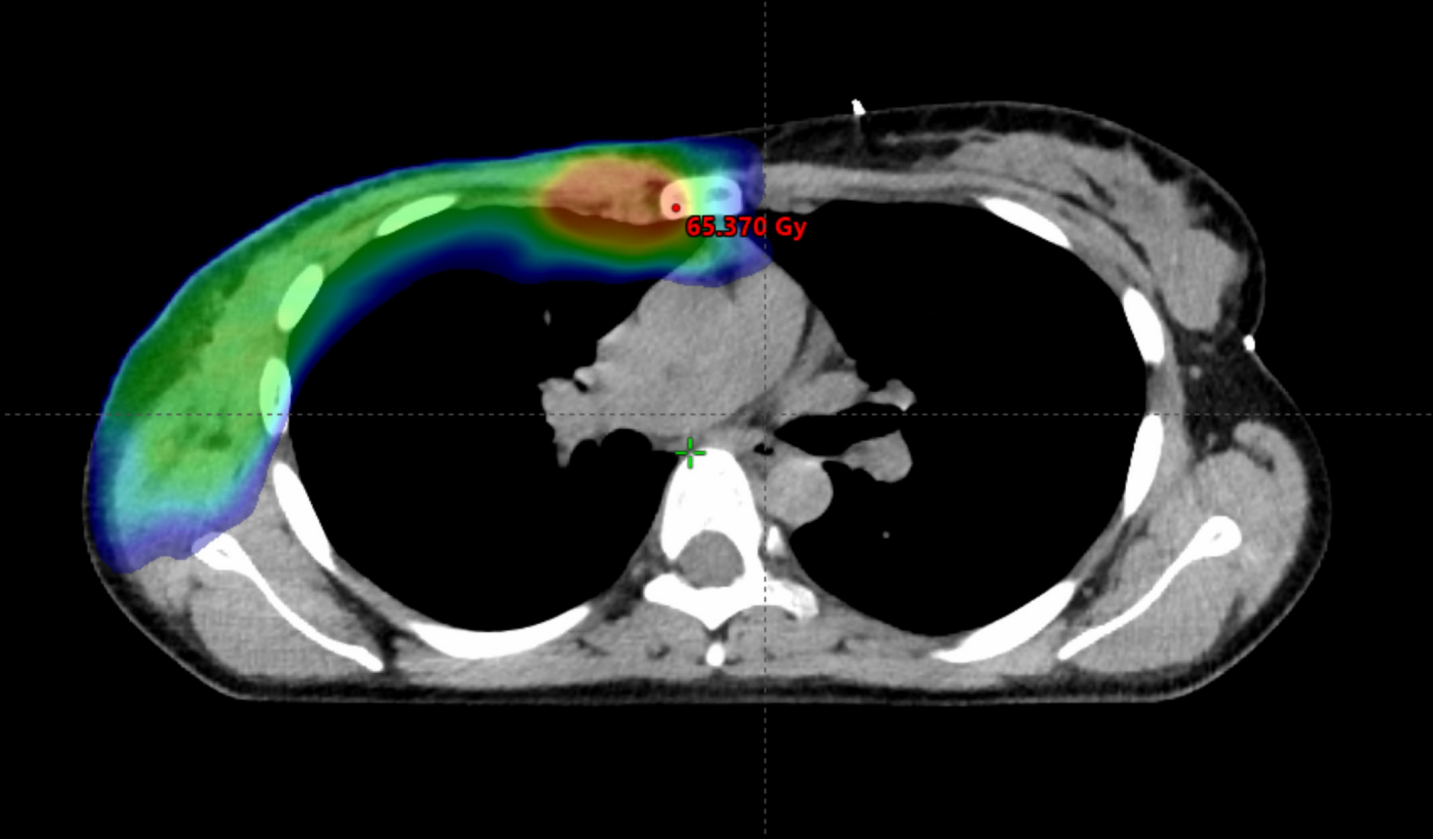
Modern radiotherapy techniques make it safe to boost the dose to the internal mammary lymph nodes. However, prior to implementing this approach, it is crucial to ascertain whether the internal mammary lymph nodes were involved and to determine the specific intercostal region affected. The irradiated volume is graphically represented, the area receiving a lower dose is in blue, while the boost area is in red.

**Figure 6:**
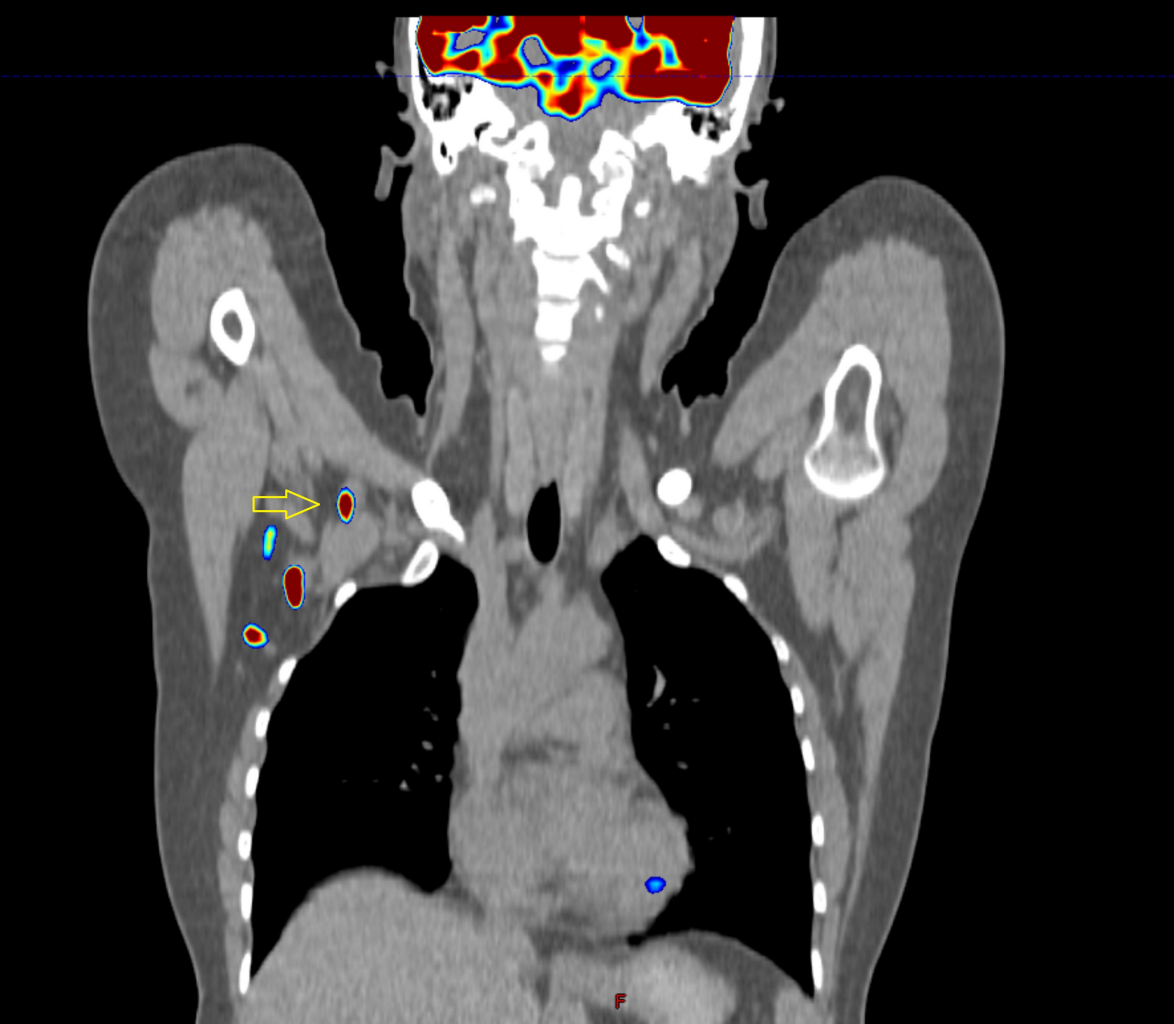
Images of a patient after axillary lymph node dissection, two out of eleven nodes contained metastases. Is this lymph node (yellow arrow) visible on baseline PET-CT operable? What has happened to the third node? It is very likely that it remained in the axilla, despite the surgeon’s efforts.

In malignancies that are highly sensitive to systemic therapy, such as nasopharyngeal cancer, it is difficult to consider modern RT planning without performing precise imaging before chemotherapy (19, 20). A thorough understanding of the original scope of the disease is required to determine the appropriate volume of irradiated tissues. The effectiveness of systemic therapy is increasing in patients with breast cancer. Regarding HER2 positive and TNBC subtypes, pCR may be expected in nearly half of the treated population, even in patients with locally advanced diseases (21, 22). The NCCN guidelines allow for a supplemental RT boost to grossly involved or enlarged lymph nodes that have not been surgically resected (23); however, there are no recommendations on how to systematically diagnose such cases clinically. PET-CT is precise in evaluating breast cancer nodal involvement and was the basis for an anatomical atlas created in 2018 (24). A study published in 2012 demonstrated that a preoperative CT scan may facilitate and increase the precision of boost planning in the tumor bed (25). Experts in the field of adjuvant radiotherapy for breast cancer suggest the possibility of using PET-CT, especially in cases where a nodal boost is relevant, or to detect additional nodes in cases of extensive nodal involvement (26). Low avidity of 2-[18F]FDG PET-CT have been noticed in slow-proliferating tumors, including invasive lobular carcinoma; however, such cases are usually not qualified for PST. The Lucerne Toolbox 2, a multidisciplinary expert consensus published in 2023 states that the radiation oncologist should review the pre-treatment images of patients undergoing PST (27). For over 10 years, we have observed the usefulness of performing PET-CT during the basic staging in cases of advanced breast cancer. Fortunately, the current European Association of Nuclear Medicine/Society of Nuclear Medicine and Molecular Imaging (EANM/SNMMI) guidelines recommend performing 2-[18F]FDG PET-CT in patients with clinical stages IIB–IV (28). Recent experience in innovative imaging modalities, such as PET-prostate-specific membrane antigen (PSMA) in patients with prostate cancer, has led to the assumption that in some cases, nodal metastases could occur at sites beyond the typical locations mentioned in current contouring guidelines (29). In terms of the radiation dose for breast cancer, hypofractionated schedules are regarded as state-of-the-art (30). However, significantly less attention has been paid to complete radiation doses. Reportedly, an increased dose to the tumor bed doubles the local effectiveness of RT; however, the boost practice varies significantly in different cancer centers (31–33). For most neoplasms, a hard-to-question paradigm in which local control depends on the total dose seems true. Modern irradiation techniques enable safe and precise application of high doses. However, it has been proven in a small group of patients with breast cancer that IMRT is effective enough to be considered as an optional radical treatment, especially in those who decline surgery (34). Data on increased doses in areas other than the tumor bed are scarce; however, using a total dose higher than 60 Gy to involved lymph nodes improves local control (35–37). Particular attention should be paid to the internal mammary and supraclavicular nodes, because these are not routinely removed during surgical procedures (38). In the 130 patients in this study, a boost dose within the involved but unoperated lymph nodes was applied in nine patients, most often 63 Gy in 28 fractions. A boost was applied to the internal mammary lymph nodes in five patients (**Figures 3**–**5**), and to the pathological lymph nodes within the part of the axilla not covered surgically in four patients (**Figure 6**). Oligometastatic disease was diagnosed in two patients, and SBRT was administered.

In the future, apart from the tumor bed boost, the dose may also be modified, depending on the risk of anatomical area involvement. Such an approach is implemented in daily practice in RT for head and neck cancers, where one RT plan often involves three different doses to various areas: a gross disease dose to the volume of direct cancer infiltration, a high-risk subclinical disease dose to the areas that are the most common recurrence sites, and a low-risk dose to the elective lymph node areas (39). Similar methods may be used in the future for cases of breast cancer where high-quality imaging techniques are available.

This study has some limitations. First, it was not a randomized trial but an observational study; moreover, PET-CT may have been performed preferentially in the group of patients with an unfavorable prognosis. Therefore, it was not possible to compare the two additional imaging modalities. Second, the number of participants recruited was small; therefore, we could not analyze the impact of personalized RT on the progression-free survival (PFS) and overall survival (OS) of patients. Third, the anatomical scope of imaging modalities varied, as CT scans included the neck, chest, and upper part of the abdominal cavity, while PET-CT covered the entire body, which is a definite advantage of this technique and enables diagnosis of oligometastatic disease in some cases. On the other hand, CT scans are not time-consuming and easily available in virtually any modern radiation therapy facility. Despite these limitations, the results of our study are encouraging. The findings of this study are likely to be validated by a large, randomized, multicenter clinical trial in the future. The strength of our study is the fact that it concerns aggressive and advanced cases of breast cancer, qualified to PST. Among this group of patients, finding lymph nodes in areas not subjected to surgical treatment is highly probable.

A distinctive feature of our approach to breast cancer imaging was the use of radiation immobilization devices. This allows for very precise overlaying of the baseline and postoperative images. Modern oncology is complex; however, while performing a medical procedure, it is worth considering whether it can be helpful in conducting the next one. Manual delineation during radiation treatment planning is subject to interobserver variability (IOV). Advances in artificial intelligence (AI) and other automation techniques are becoming helpful to radiation oncologists and radiologists. Moreover, studies have shown that these systems can be effective, provided the input data is of high quality (40, 41).

## 5 Conclusions

Our results indicate that both CT and PET-CT enable a detailed assessment of the location and size of primary tumors in the breast as well as pathological lymph nodes. Unlike MRI, which is most often performed in the prone position, which significantly affects anatomical features, both CT and PET-CT scans may be performed in the same layout as the therapeutic position. Furthermore, numerous researchers recommend performing CT or PET-CT in cases of suspected spread of the disease (42, 43). However, considering the capabilities of modern RT, routine and accurate imaging should be performed in a systematic way. In our opinion, adequate patient selection for preoperative systemic therapy should be performed by a MDT. This group of patients will benefit the most from extended radiological staging.

## Data Availability

The raw data supporting the conclusions of this article will be made available by the authors, without undue reservation.
