# Supplementary material for: Baseline cross-sectional imaging of locally advanced high-risk breast cancer facilitates highly customized radiation therapy in surgically inaccessible anatomical areas
Source: Front Oncol. 2025 Feb 27;15:1556122. doi: 10.3389/fonc.2025.1556122 (PMC11923551; doi:10.3389/fonc.2025.1556122)
Supplement: Supplementary file 1 [file DataSheet1.docx]

Supplementary Material

Baseline cross-sectional imaging of locally advanced high-risk breast cancer facilitates highly customized radiation therapy in surgically inaccessible anatomical areas

**Abbreviations:**

**CT scan** (computed tomography scan, performed using intravenous contrast agent), **LDCT** (low-dose CT, a component of the PET-CT scan), **MMG** (mammography), **MRI** (magnetic resonance imaging), **PET-CT** (positron emission tomography), **planning CT** (non-contrast-enhanced computer tomography, performed in supine treatment position), **US** (ultrasound),

**MEDICAL IMAGING IN BREAST CANCER - SHORT OVERVIEW**

During MMG (mammography) breast is compressed, which changes its shape to a certain degree. This imaging modality is not reliable in level III and IV axillary lymph node assessment. The advantage of MMG is the possibility of verification by a breast radiologist at a reference center, usually without the need to repeat the procedure. Quality of US (ultrasound) depends on experience of performing physician. Pressure exerted by the transducer can deform the breast to some extent, which may be important in evaluating the distance between tumour and skin. Only the scan description is of use for a radiation oncologist. In case of doubt, the entire procedure needs to be repeated at a reference center. Evaluation of internal mammary lymph nodes also appears to be challenging. MRI (magnetic resonance imaging) is a modern imaging technique with limited availability in many regions of the world. Another issue is the prone position of the patient during the procedure. While MRI images can be uploaded to the radiotherapy planning system, achieving a satisfactory fusion is difficult due to the completely different shape of the sagging breast and the altered shoulder position (Fig. 1, Fig.2), , compared to the supine position typically used in radiotherapy planning.


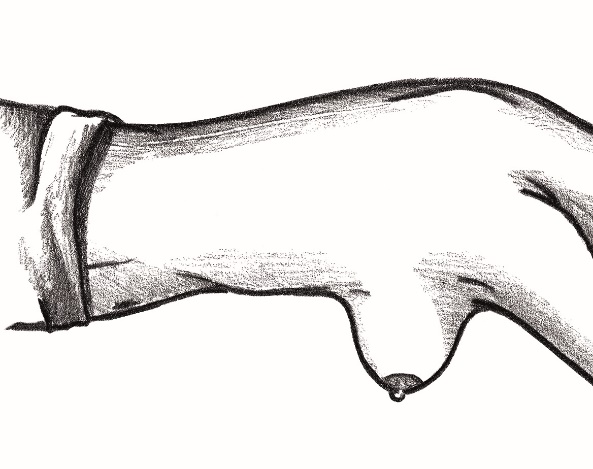

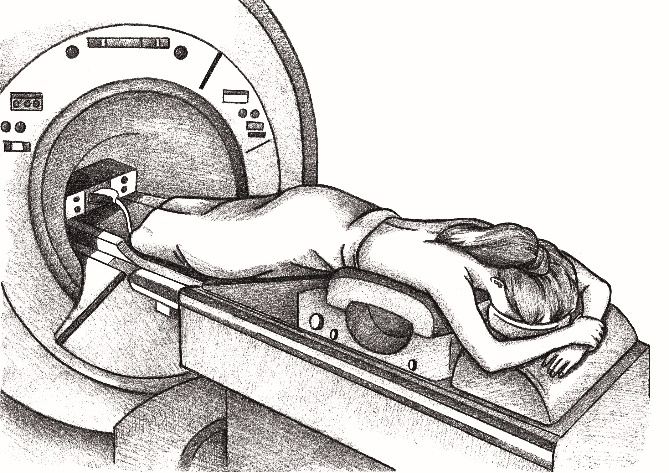


Fig. 1 Fig. 2

**EXTENDED RADIOLOGICAL STAGING**

Orfit-AIO system was used for positioning. Layout (Fig. 3) can be customized based on various factors such as anatomical conditions, breast size, and the patient’s fitness. Most of the sets enable the selection of appropriate back support and adjustment of the location of the shoulder immobilization device. The positional information can be easily stored for future reference, allowing for the recreation of the same body position during subsequent CT scans or PET-CT.


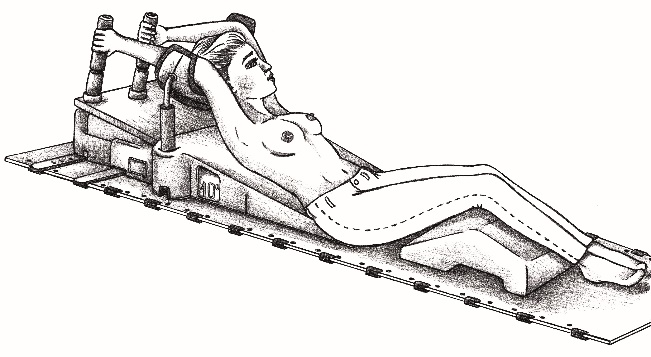
 Fig. 3


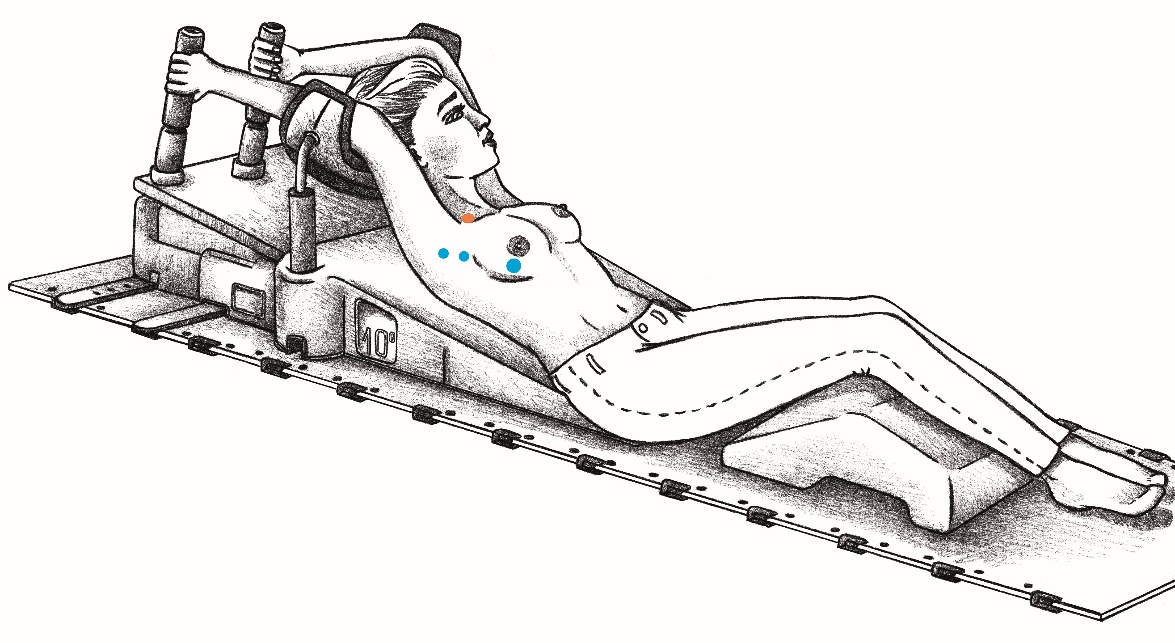


Fig. 4

CT scan or PET-CT conducted prior to systemic therapy provides precise imaging of the disease. The blue dots depicted on the Fig. 4 represents cancer foci within the breast and lymph nodes in the axillary fossa, which are the areas subjected to surgery, while the range dot represents a pathological lymph node in an area that has not been operated on.


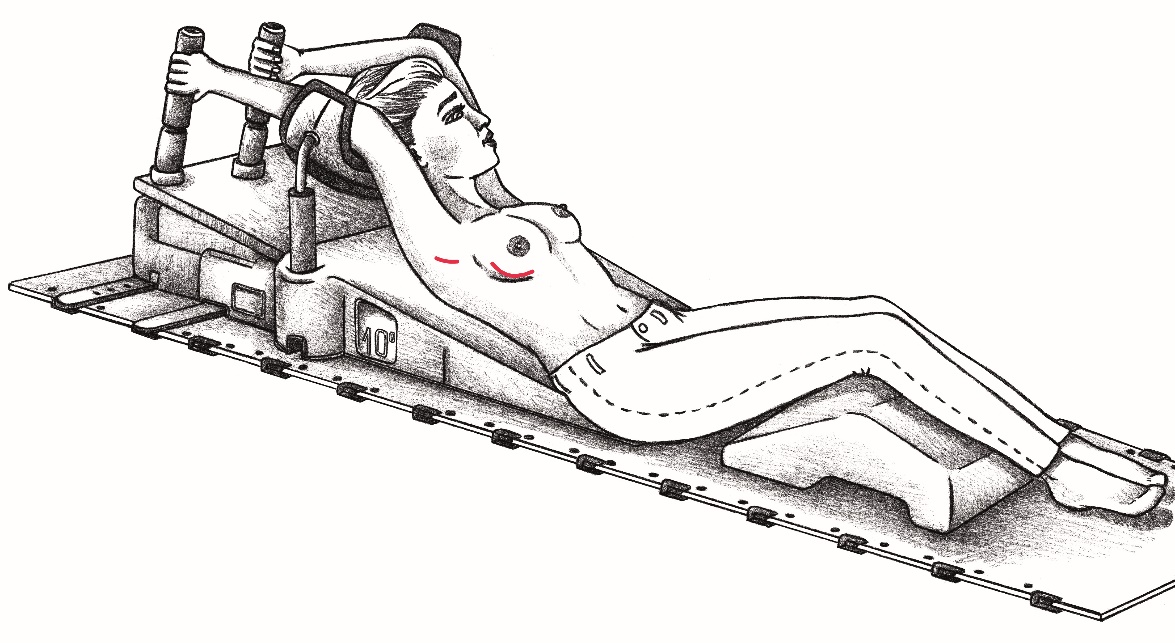


Fig. 5

A routine planning CT is performed after systemic therapy and surgery. In most patients, only the effects of surgery may be visible: red lines reflect the presence of surgical scar, tracers left in the tumor bed, and sometimes the occurrence of seroma or axillary lymphocele (Fig. 5). The lymph node in the supraclavicular region, which was previously exposed to systemic therapy, may be in complete remission and therefore not visible on the planning CT.


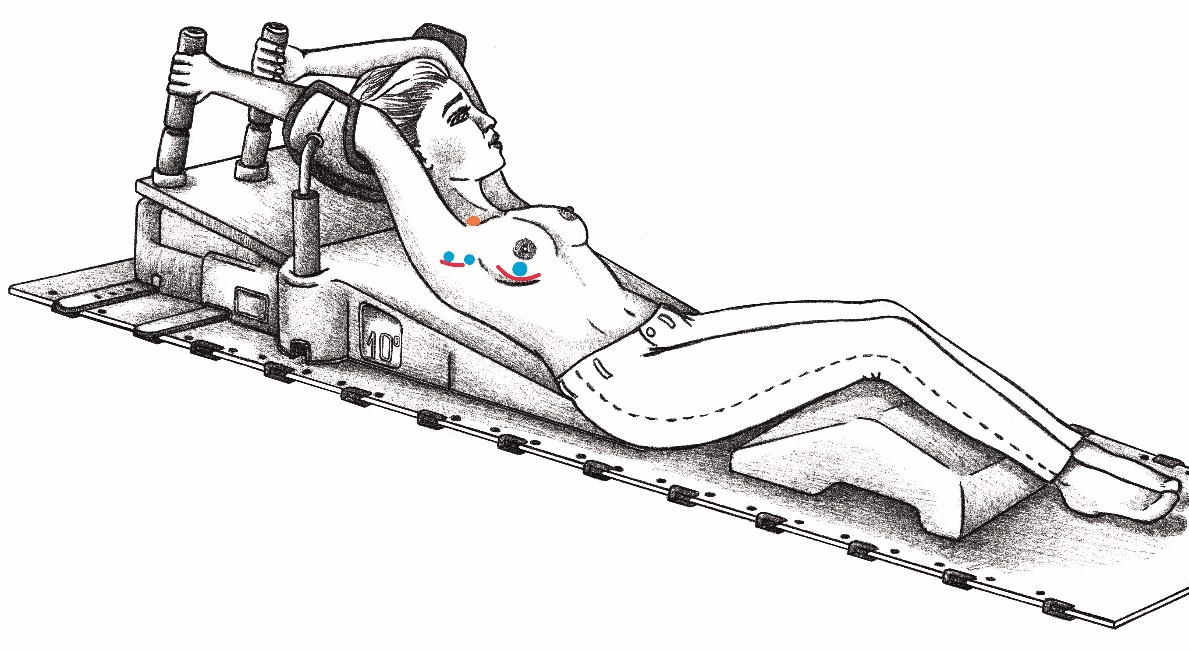


Fig. 6

Medical imaging in a therapeutic position before systemic therapy and surgery, followed by routine planning CT, enables image fusion. This approach offers numerous possibilities for radiotherapy customization, such as increased dose in the non-operated anatomical area, where the pathological lymph nodes were observed (e.g. supraclavicular region or internal mammary lymph nodes). Additionally, it can aid in the diagnosis of oligometastatic disease and facilitate the application of stereotactic body radiotherapy (SBRT).

**SAMPLE IMAGES**

The images have been grouped into five categories:

1. Primary tumor focus in the breast.
2. Lymph nodes in the axillary fossa.
3. Involved lymph nodes outside the surgeons’ reach.
4. Oligometastatic disease.
5. Polymetastatic disease.

Both CT scan and PET-CT enable a comprehensive evaluation of the primary tumor focus in the breast, including its location and size, as well as the presence and location of pathological lymph nodes. This additional diagnostic information adds value to the standard initial workup. In certain cases, personalized radiotherapy (boost within the internal mammary lymph nodes, stereotactic body radiotherapy in oligometastatic disease) was possible to carry out.


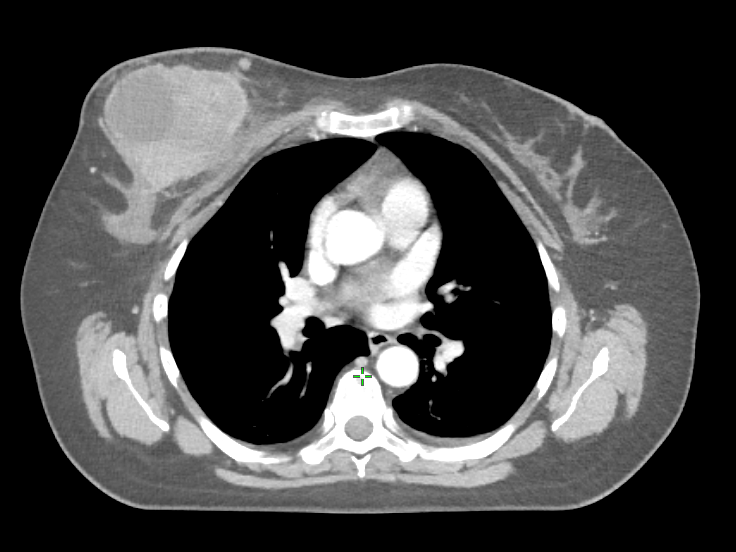
**1.** **Primary tumor focus in the breast.**

Img. 1.1

CT image of cT3N0M0 TNBC, in the right breast (Img. 1.1), next to a massive lesion, another small lesion is visible, which may be a satellite focus.


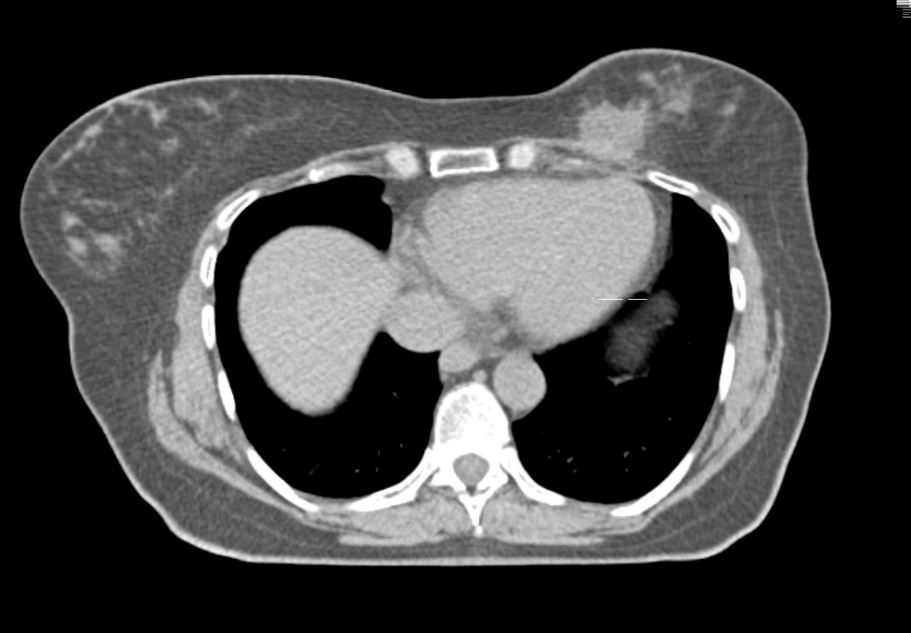


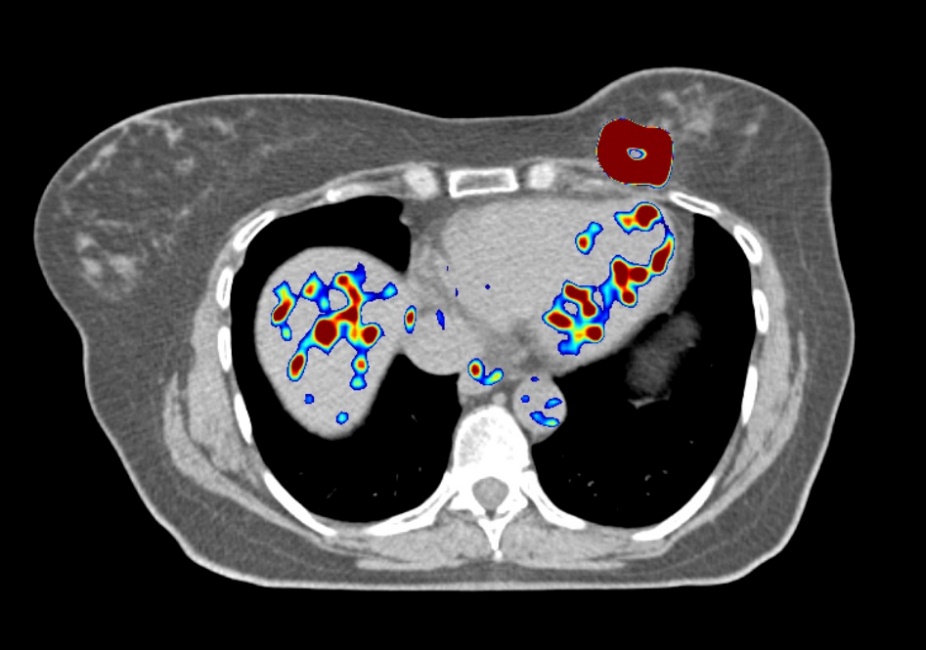
Img. 1.2

Img. 1.3

The PET-CT fusion image (Img. 1.3) and LDCT (Img. 1.2) of the patient with TNBC of the left breast, which suggested infiltration of the pectoral muscle, despite the tumor's relatively small size. Unfortunately, this suspicion was confirmed by the clinical course of the disease. Two years after completing the primary treatment, the cancer has recurred in the chest wall.


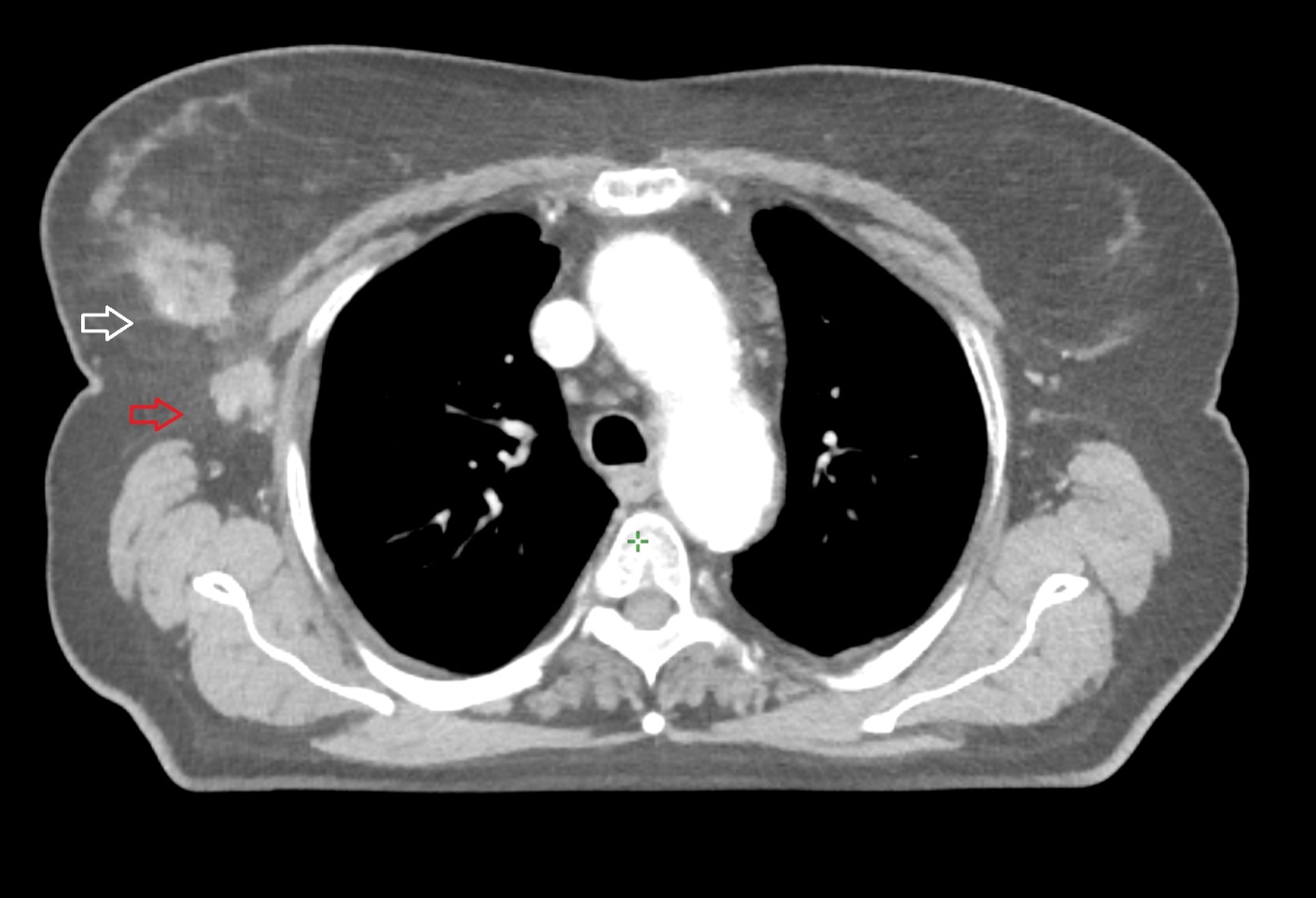


Img. 1.4

The CT image of cT3N1 Luminal B cancer of the right breast (Img. 1.4), both primary focus (white arrow) and pathological axillary nodes (red arrow) are clearly visible. The CT scan allowed the diagnosis of multiple metastases in the mediastinal lymph nodes in this patient.

**2.** **Lymph nodes in the axillary fossa.**
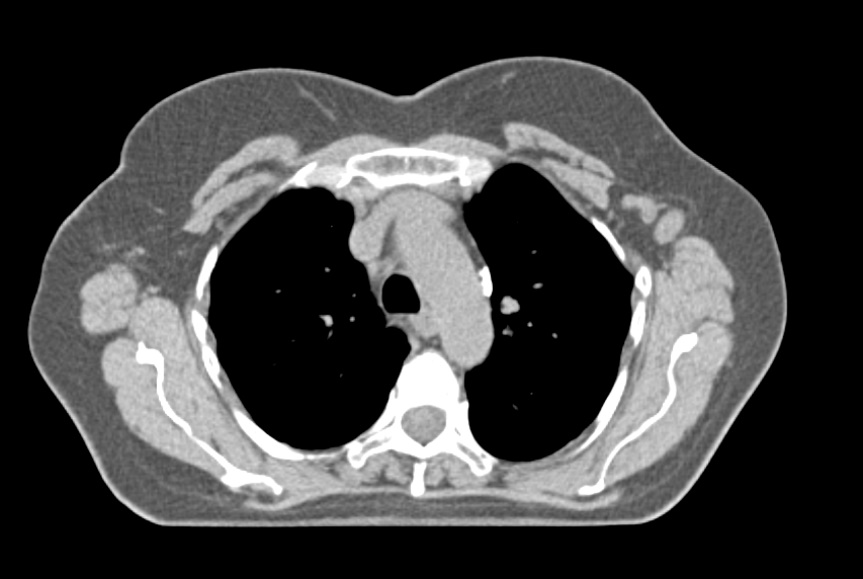


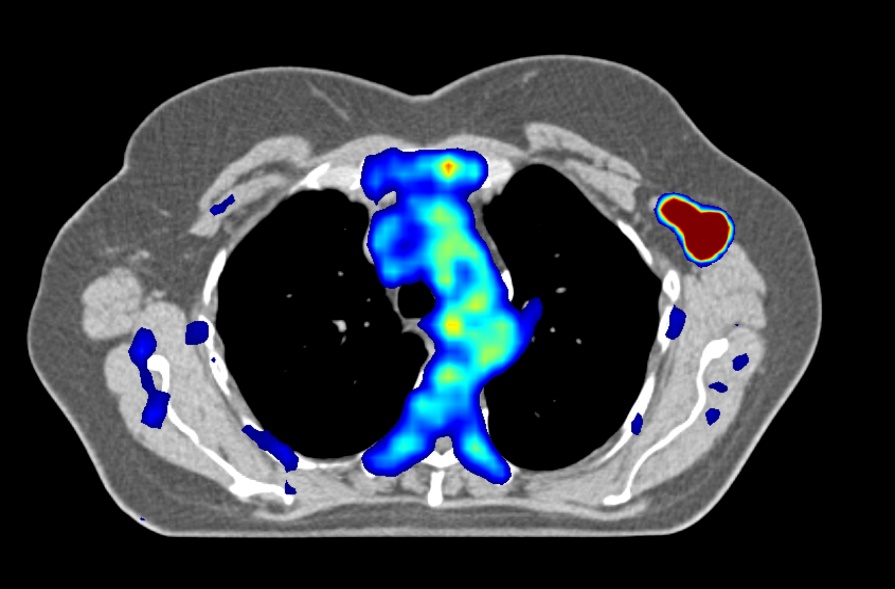
Img. 2.1

Img. 2.2

The PET-CT fusion image (Img. 2.2) and LDCT (Img. 2.1) of the patient with cT2N3 Luminal B cancer of the left breast. In addition to visualizing the primary lesion and lymph nodes, the PET examination also showed numerous metastases in the bones.

**3. Involved lymph nodes outside the surgeons’ reach.**


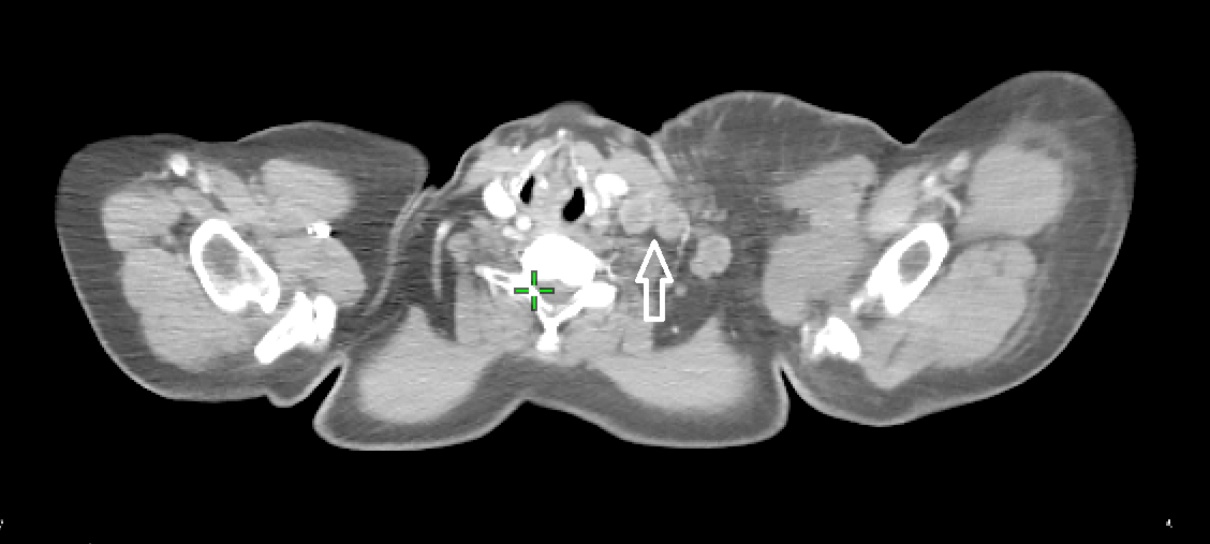


Img. 3.1

In some cases, there are nodal metastases occupying both the supraclavicular area and the upper levels of the neck (Img. 3.1). In the absence of distant metastases, they can be treated with radical radiotherapy. However, it is crucial to rule out synchronous head and neck cancer. In this case, pathological lymph nodes at the level of the larynx were visualized (white arrow).


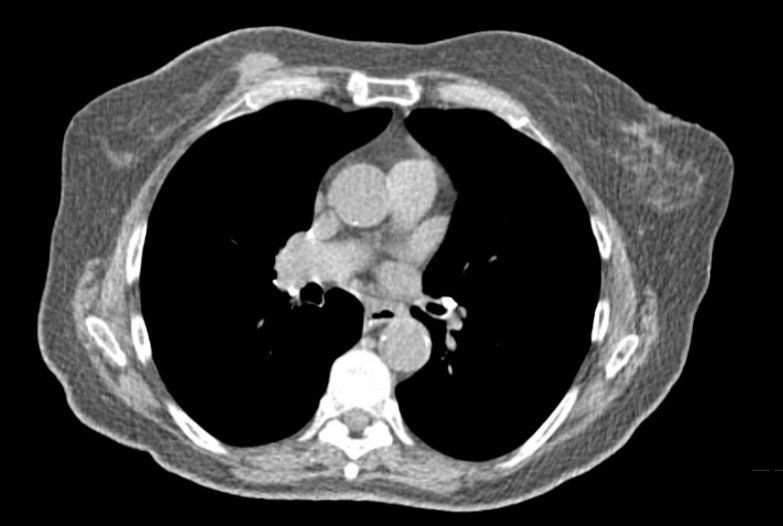

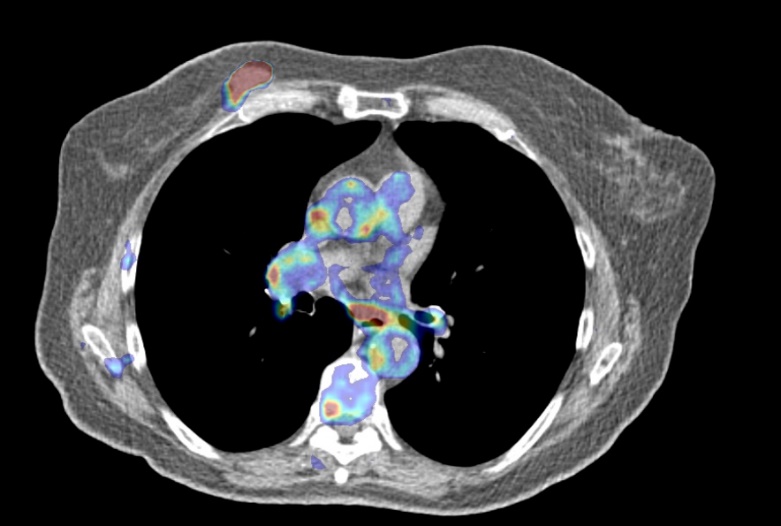
**4. Oligometastatic disease.**

Img. 4.1. Img. 4.2


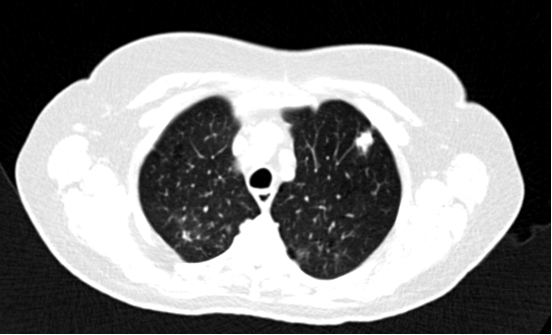
The PET-CT fusion image (Img. 4.2) and LDCT (Img. 4.1) of the patient with cT2N0 Luminal B-like, Her2-positive cancer of the right breast. Additional imaging revealed the presence of two metastatic nodules in the lungs, that could be treated with ablative doses of SBRT. The images below show the nodule in the left lung (Img. 4.3, Img. 4.4), and the estimated dose distribution achieved during the treatment (Img. 4.5).

Img. 4.3

The patient received 4 x 12 Gy within the lung lesions, the image shows a hot spot in the nodule where the total dose exceeded 60 Gy (Img. 4.5). Long-term disease control was achieved.


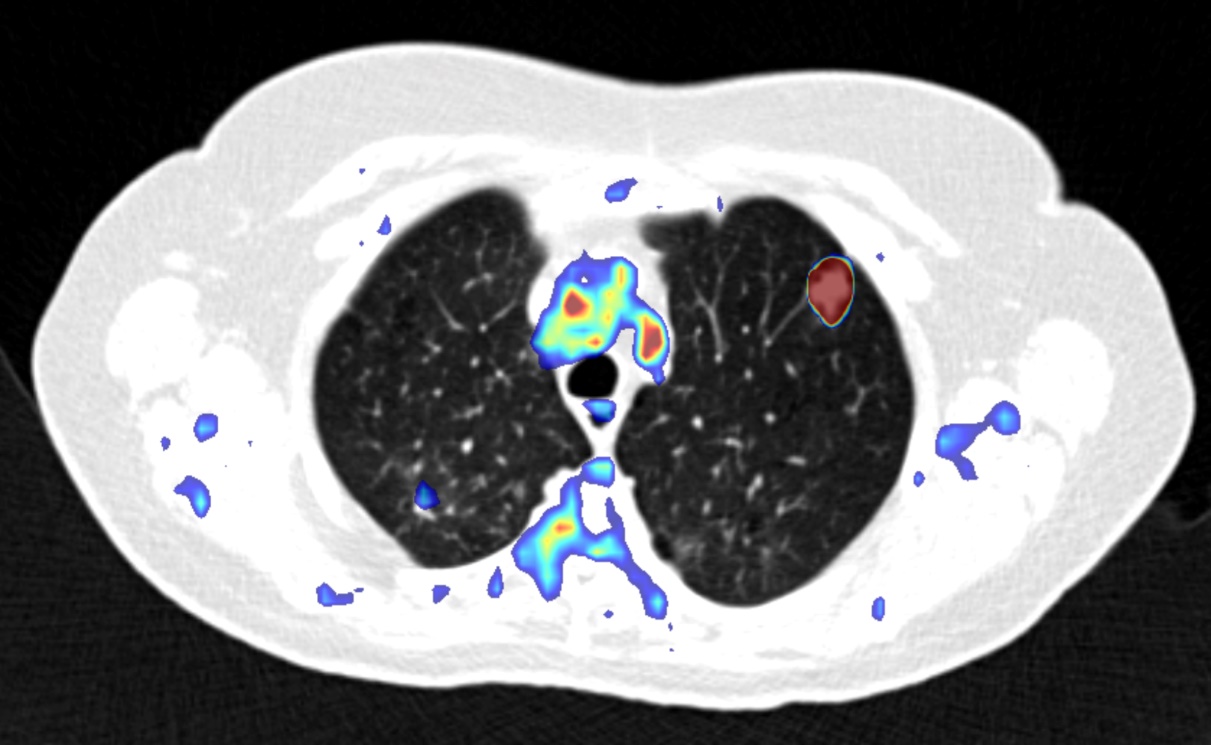


Img. 4.4


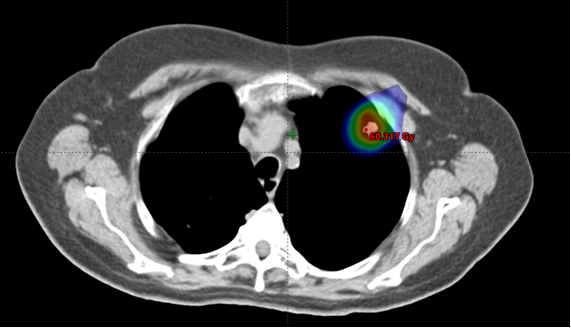


Img. 4.5

**5. Polymetastatic disease.**


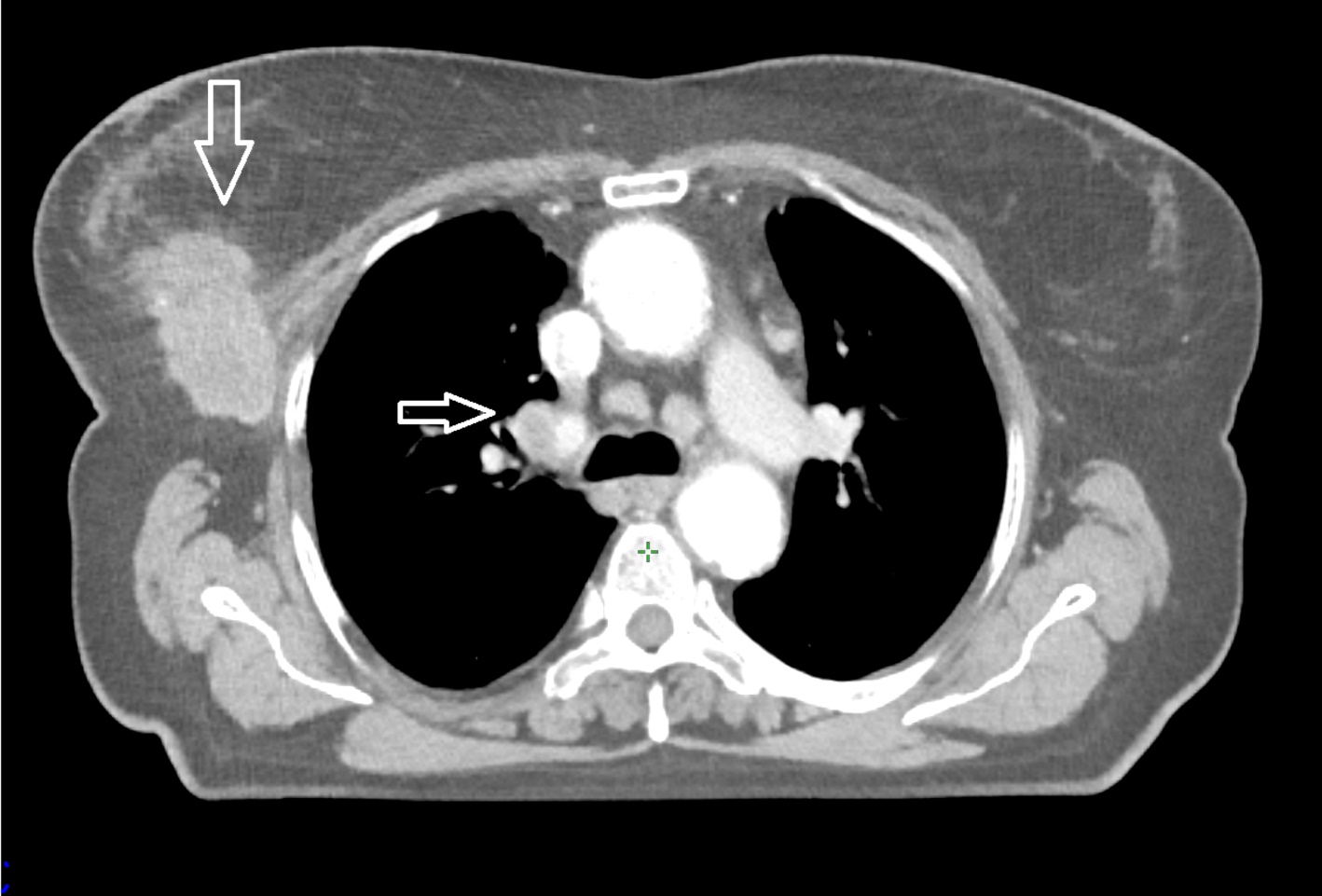


Img. 5.1

The CT image of cT3N1 Luminal B cancer of the right breast, representing the same patient as depicted in Img. 1.4. The CT scan revealed the involvement of mediastinal lymph nodes. An additional examination allowed to make a rational clinical decision; radical treatment was abandoned. At the time of diagnosis, the patient was 75 years old. Three years later, she had a stroke, successfully treated with thrombolysis; several years later she suffered a heart attack, requiring several months of rehabilitation. During a visit to the Breast Unit seven years after the breast cancer diagnosis, the patient was 82 years old, and her performance status was estimated as ECOG – 1. The cancer has been controlled for all these years by systemic treatment based mainly on endocrine therapy.


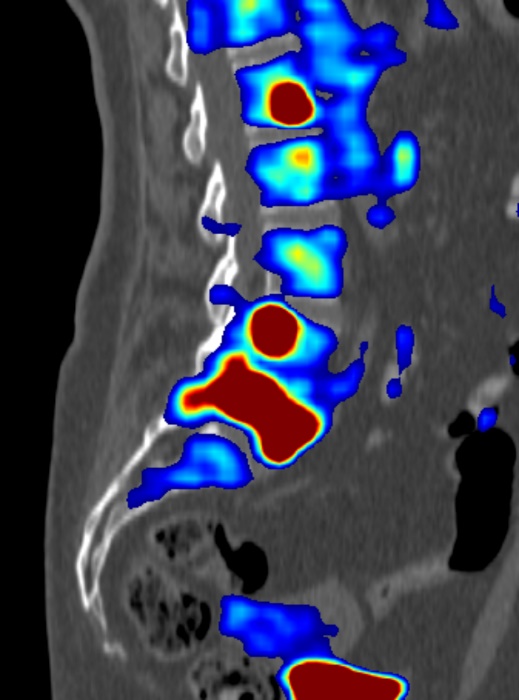

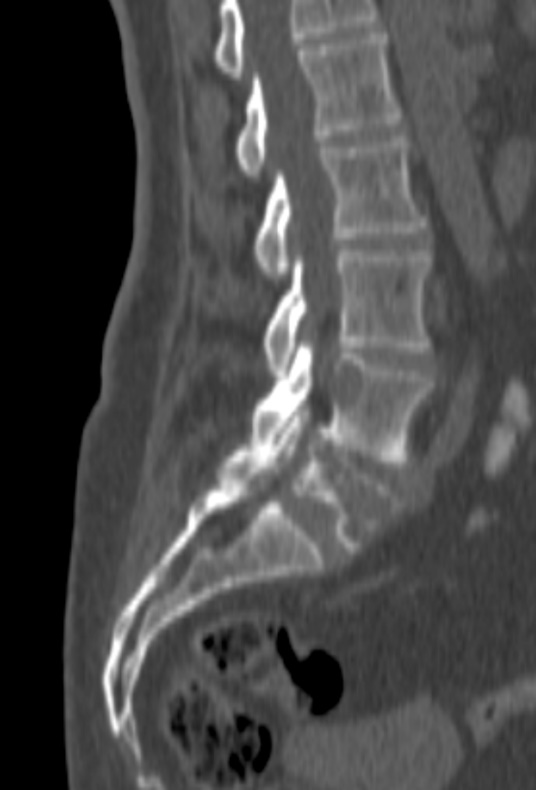


Img. 5.2 Img. 5.3.

The PET-CT fusion image (Img. 5.3) and LDCT (Img. 5.2) of the patient with cT2N3 Luminal B cancer of the left breast. In addition to visualizing the primary lesion and lymph nodes, the PET examination revealed numerous metastases in the bones. It is noteworthy that this patient is the same individual as depicted in Img. 2.1 and Img. 2.2, where attention was focused on the axillary fossa.

**
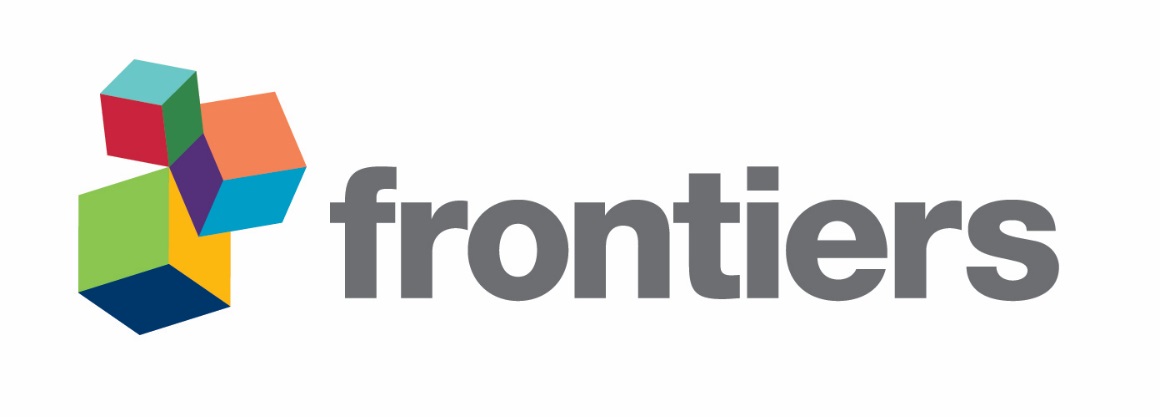
**
